# Perspectives from health, social care and policy stakeholders on the value of a single self-report outcome measure across long-term conditions: a qualitative study

**DOI:** 10.1136/bmjopen-2014-006986

**Published:** 2015-05-19

**Authors:** Cheryl Hunter, Ray Fitzpatrick, Crispin Jenkinson, Anne-Sophie Emma Darlington, Angela Coulter, Julien E Forder, Michele Peters

**Affiliations:** 1 Academic Unit of Primary Care, Leeds Institute of Health Sciences, University of Leeds, Leeds, England; 2 Health Services Research Unit, Nuffield Department of Population Health, University of Oxford, Oxford, England; 3 Faculty of Health Sciences, University of Southampton, Southampton, England; 4 PSSRU, University of Kent, Canterbury, England; 5 PSSRU, London School of Economics and Political Science, London, England

**Keywords:** PRIMARY CARE, QUALITATIVE RESEARCH

## Abstract

**Objectives:**

To explore the views of a range of stakeholders regarding whether patient-reported outcome measures (PROMs) can be developed to measure key attributes of long-term conditions (LTCs) care in England, and the potential value of a single generic measure.

**Design:**

Qualitative semistructured interview study, analysed using a framework approach.

**Participants and setting:**

Interviews with 31 stakeholders from primary care, secondary care, social care, policy and patient-focused voluntary organisations in England.

**Results:**

There was broad support for a single PROM that could be used to measure outcomes for patients with any LTCs in any health or social care setting. Interviewees identified three desired uses for a PROM: to improve the quality of individual care; to increase people's engagement in their own care; and to monitor the performance of services. Interviewees felt that a PROM for LTCs should incorporate a mixture of traditional and non-traditional domains, such as functioning, empowerment and social participation, and be codesigned with patients and professional end-users. Stakeholders emphasised the need for a PROM to be feasible for practical implementation at the individual clinical level as a first priority. A number of concerns and potential problems were identified in relation to the application and interpretation of an LTC PROM.

**Conclusions:**

This study has demonstrated support for a single self-report outcome measure that reflects the priorities of people with LTCs, if such a measure can be shown to be meaningful and useful at the individual level. People with LTCs and professional end-users in health and social care should be involved in the development and evaluation of such a measure.

**Strengths and limitations of this study**

- This study incorporates a wide range of perspectives on the potential value of a patient-reported outcome measure (PROM) for long-term conditions (LTCs) from across health, social care and voluntary organisations and from managerial, policy and front-line levels.
- The findings offer support for the idea of a single measure that can work across LTCs and be used to improve care for people with LTCs.
- Several domains were identified for an LTC PROM, including traditional domains such as quality of life, functioning and social participation and less traditional domains such as empowerment and support from services.
- A limitation of the study is that it focused on health and social care context in England; however, the issues identified are likely to be applicable across countries.
- The study involved purposive and snowball sampling, focusing on those with experience of PROMs and LTCs, and there may be some bias towards including like-minded stakeholders.

## Introduction

Long-term conditions (LTCs) pose an enormous challenge to healthcare systems because of their prevalence and complexity, exacerbated by an increase in the number of people living with multimorbidities.^1^ ^2^ It is argued that the scale of this challenge requires major system-level changes.^3^ Greater patient engagement, improved self-management support and individualised care, are seen as key elements in policies designed to improve care for LTCs.^4–9^

Patient-reported outcome measures (PROMs) have been proposed as a technology that may strengthen patient engagement and enable individualisation of care.^10^ PROMs were initially developed to enable outcome measurement in clinical trials to take account of people's subjective health status and health-related quality of life.^11^ Disease-specific and generic PROMs exist for use with people with LTCs, and these offer different benefits and limitations. For instance, disease-specific PROMs tend to be more sensitive to change, but can only be used in a specific population. In order to compare across LTCs, or capture outcomes for multiple LTCs, a generic PROM would be required but might be less relatable to patients’ specific needs and contexts due to its broad scope.^10^ More recently, their use has been promoted and evaluated for other contexts and applications. At the individual level, PROMs have been promoted as a means of improving communication between patients and healthcare professionals; assessing effectiveness of treatments; influencing clinical management; enhancing patient involvement, health behaviours and satisfaction with services; and improving detection and monitoring of symptoms.^11–15^ Results to date have been mixed; while patients and practitioners tend to respond positively to the idea of PROMs, the impact of PROMs on clinical practice has been equivocal.^16^ ^17^

An alternative role for PROMs is in providing aggregated evidence of the performance and quality of services.^18^ Several healthcare systems worldwide have implemented the routine collection of PROM data, one example being the English National Health Service (NHS), which has collected data before and after elective surgeries since 2009.^19–22^ Studies have demonstrated that collecting PROM data in this way is feasible, and extending the routine collection of PROM data to LTCs has been advocated.^21–23^ However, the issue of using PROMs to evaluate service performance in relation to LTCs is challenging,^24^ and evidence for the impact of PROMs on service improvement is weak.^16^ ^17^ ^25^ ^26^ In England, the standardised health status measure EQ-5D has been included in an annual population-based survey of primary care (the GP Patient Survey) and a primary care pilot study to assess feasibility of regular monitoring of health-related quality of life in people with LTCs.^23^ ^27^ Response rates have been low for these surveys,^23^ ^28^ and the pilot study raised questions about the suitability of the EQ-5D to detect change in LTCs within the primary care population over time.^29^ Studies suggest that further work is needed to determine how to collect PROM data most effectively, and how to ensure implementation of PROM data for service improvement purposes.

A further challenge in relation to PROMs for LTCs is whether PROMs can usefully contribute to the integration of health and social care services. LTCs are most common in the ageing population, where needs often extend beyond the medical.^30^ ^31^ Social care in the UK refers to a range of care activities oriented around providing for people's basic daily needs (such as dressing and feeding), as well as their social and emotional needs.^32^ Those with complex needs are most likely to experience fragmentation, poorer quality of services and poorer health outcomes.^33–37^ It is argued that integration can improve the quality of care by reducing fragmentation, facilitating a more patient-centred approach to care and improving access and communication across services.^38^ Whether a PROM can inform and contribute to integration is open for debate.

Alongside PROMs, there has been a rise in interest in Patient-Reported Experience Measures (PREMs).^39^ Capturing patient experience is also a priority in the UK NHS context, directed at improving the quality of care.^40^ PREMs tend to be surveys that aim to capture patient experiences of care in a systematic way, although there are other methods of capturing experience that are more individualised and debates around the best ways to collect and use patient experience data.^41^

Despite limited evidence of the impact of PROMs either at the individual or aggregate and system level, considerable interest exists in their potential to positively impact on the quality of LTC care.^10^ ^13^ Previous research has highlighted the importance of stakeholder engagement when designing and implementing PROMs, yet no studies to date have examined the views of the range of stakeholders potentially involved in integrated LTC care. We therefore undertook a study of stakeholders from health, social care and community services to determine their views on the likely value of a PROM designed specifically for use with people with LTCs. Below, we have used the term ‘patient’ to refer to people with an LTC accessing any service, and the term ‘PROMs’ to refer to self-report measures for use by people with LTCs across the health and social care system.

## Methods

We conducted semistructured qualitative interviews with stakeholders in health, social care and community services, including commissioners, policymakers, service providers, health and social care services managers, front-line clinicians and patient-focused voluntary organisations.

The semistructured topic guide was informed by current literature on PROMs, in particular healthcare and social care policy documents.^4–9^ ^31^ ^42^ It was codeveloped by the authors, who have extensive experience of developing and working with PROMs. Questions focused on the participant's current role and interests regarding PROMs and LTCs; uses for an LTC PROM; settings for the use of an LTC PROM; users and beneficiaries of PROMs and PROM data; concerns around and issues with the use of an LTC PROM and PROM data; and suggestions of PROM content relevant to LTCs in health and social care. Participants were recruited for their experience and expertise around LTCs and PROMs, and were asked to summarise their experience at the beginning of the interviews. Regarding potential PROM content, participants were initially asked an open question, followed by prompts to consider particular areas, with most participants referring to existing PROMs or PREMs with which they were familiar.

Participants were recruited through a combination of purposive and snowball sampling. The interviewers (ASD and CH) contacted people who were known to have an interest in LTCs and/or PROMs. Interviewees often referred the interviewers to other relevant stakeholders. Interviews were audio recorded following informed consent, and transcribed verbatim by a professional transcriber. Data collection continued until saturation of themes relevant to a PROM for LTCs had been reached.

Initial thematic analysis was iteratively carried out during data collection, and was used to develop a thematic framework (see box 1); QSR NVivo 10 was used to manage data and complete the analyses using a framework approach.^43^ The analyses were led by CH, ASD, RF and MP analysed a subset of transcripts to confirm the main themes (ASD and MP reviewed five and RF reviewed six transcripts). Themes were discussed and refined within the research team.

Box 1 **Initial thematic framework**

- Background to PROMs and LTCs
- Content of PROMs
  Experience or Process domains
  Inter-relationships
  Outcome domains
- Defining PROMs
- Designing an LTC PROM
  Process of designing an LTC PROM
  Existing models of PROMs
  Defining aims for an LTC PROM
- Problems with or for PROMs
  Systemic
  Cultural
  Patient-specific
  Service-specific
  Implementation
  Interpretation
  LTC-specific
  Measurement-specific
- Uses of PROMs
  Commissioning
  Provider performance
  Quality improvement
  Patient-specific
  LTC-specific
  Service-specific
  Cross-service uses

Ethical approval was granted by the Central University Research Ethics Committee (CUREC) at the University of Oxford (Reference Number: MSD-IDREC-C1-2013-206, 2 December 2013).

## Results

### Sample characteristics

Twenty-nine interviews were completed, with 31 participants (see table 1). Two interviews were joint interviews, involving two participants. All but two interviews were completed by telephone; these two interviews were conducted face-to-face. Interviews lasted on average 40 min (range: 10–79 min).

**Table 1:** Summary of participants

Job role	Number of participants with job role
NHS policy and commissioning	4
Health and social care service regulator	1
Front-line clinician	
GP	4
Nurse practitioner	1
Psychiatrist	1
GP commissioner	3
Consultant physician	2
Social care services manager	3
Voluntary organisation	6
Healthcare service provider	3
Clinical commissioning group non-clinical members	2
Public health commissioning	2
Patient and public involvement representative	1
Total number of participants interviewed (NB: Two participants held more than one job role, and are counted twice in the table, but once in the total)	31

GP, general practitioner; NHS, National Health Service.

Based on participant self-report, it seemed that all front-line clinicians had direct experience of working with people with LTCs, but typically described limited experience of using PROMs in practice or in research. Those with a policy or commissioning role demonstrated more extensive knowledge of PROMs. The voluntary organisation participants worked with different groups, including people with cancer, mental health problems, multiple LTCs and social care needs. They tended to indicate high levels of knowledge and experience of PROMs. Participants from social care described themselves as knowledgeable about PROMs due to their inclusion in annual social care surveys, but were less used to categorising service users by diagnostic labels, as people are usually identified by need rather than diagnosis in social care practice.

### Findings

We report findings under three main themes, as identified through thematic analysis of the transcripts, and utilised in the thematic framework (box 1). The subthemes highlight the most salient issues and areas of variation across stakeholders.

### Theme 1: uses and users of an LTC PROM

Participants talked about several uses that they foresaw or desired for an LTC PROM, including using a PROM to: improve care through informing the re-design of services; improve care through influencing the conversation between patient and practitioner; promote patient involvement in their own care; and monitor the outcomes of interventions. Participants debated whether it would be possible to use the same PROM to achieve multiple purposes. Underlying the proposed uses was a consensus that LTC care needs to be re-oriented to prioritise a holistic and patient-driven approach to care, which aims to support people to maintain a desired level of functioning and quality of life, and to enable people to manage their own LTCs independently (table 2).

**Table 2:** Theme 1—uses and users of an LTC PROM

Theme	Subthemes	Examples
Uses and users of an LTC PROM	A tool for improving care—through re-designed services	You could hold all the providers involved in that long-term condition (…) to account for what you're sort of getting back in the PROM (P3, NHS Policy and Commissioning)
	*Users: Practitioners, Individual Services, Provider Groups, Commissioners*	A system that is more focused around the patient and a contracting methodology that supports organisations to do that and aligns incentives, would also be a more cost effective system that gives us better value (…) for patients, the services and the tax payer (P13, CCG Manager)
	A tool for improving care—through informing the patient—practitioner conversation	If [the PROM] becomes about changing the way that a patient is using their consultation, the relationship with the doctor, and making those people listen to each other and think about what the patient wants then that would be good (P1, GP)
	*Users: Patients, Practitioners, Individual Services*	If we're measuring ‘doing to [patients]’, that promotes doing to and people will keep doing to. If we're measuring ‘doing with and working with [patients]’, that will promote doing with and working with (P17, Consultant)
		What you're really aiming to do [in clinical practice] is optimise concordance between doctors and patients—you're trying to align what they're both after and I think that the tool [the PROM], the thing I would find it really useful for is a relatively swift way of getting to what the patient really thinks (P29, GP)
	A means of involving people in their own care	There is a role for service users and patients and those in receipt of services actually using tools of this sort to affect, influence and shape services for their benefit (P15, PPI)
	*Users: Patients, Practitioners, Individual Services*	If you have an instrument that gives, measures [a patient] with a score and you can feed back the score then I think that can have a very positive effect on empowerment (P22, GP)
		I would use [a PROM] for a patient to reflect on how their condition has been over the last two years (…) you'd probably need to tie it to something; that something that they did at regular intervals (…) you'd probably want to have it as a tool to use as opposed to something that had to be done (P29, GP)
	Capturing the outcomes of interventions	The important thing is to be clear about what you're putting in, i.e. the intervention (…) [and] be sure that the measurement is connected to that. (…) [C]are planning is a process but it's a specific intervention (…) so what I would like to see is a PROM that measures the outcomes of the care planning (P10, Consultant)
	*Users: Patients, Practitioners, Individual Services, Provider Groups, Commissioners*	It seems to me that if the future direction [of healthcare] is to have a model of care that is more than medicine, that's built around personalised care planning and that, you know, is all about enabling people to manage their lives and conditions as successfully as possible (…) then a PROM type measure that could be used on a regular basis by, you know, by the person and the key professionals co-ordinating their care (…) would have potential value (P20, Voluntary Organisation)
	Using a PROM for multiple purposes	[To use one measure for multiple purposes] feels very complex but at the same time one would hope that you'd be able to align them all so that you're not using different things with people (…) I think that there is a place to be doing it individually but also [at a] population [level] (P 6, GP and CCG)
	*Users: Patients, Practitioners, Individual Services, Provider Groups, Commissioners*	

GP, general practitioner; LTC PROM, long-term conditions patient-reported outcome measures; NHS, National Health Service.

#### A tool for improving care—through re-designed services

One of the main uses proposed for an LTC PROM was that it could be used to monitor services and align different providers to the same outcomes. The idea underlying this use was that re-designing and incentivising services to achieve the same outcomes: would promote integration by encouraging a sense of shared responsibility across services; would reduce duplication and fragmentation of effort; and would enable more effective patient-driven care. The content of the LTC PROM would thus represent shared outcomes across services, and PROM data would be used to measure success towards achieving these outcomes.

Multiple users were envisaged for data generated in this manner. Interviewees felt that this could be an important source of data to inform commissioning and service provision decisions, and to hold providers to account for achieving valued outcomes. It was also felt that this use could inform practice at the individual level.

#### A tool for improving care—through informing patient–practitioner conversation

Most participants saw a PROM as a tool that could be used at the level of the patient–practitioner interaction. The PROM would be used to open up a conversation about outcomes and needs, and then inform health and/or care decisions. In this use, both patient and practitioner were envisaged as active participants in decision-making and in performing actions arising from the conversation. It was also felt that using an explicit tool such as a PROM to guide the conversation would enhance concordance regarding decisions made.

This was envisaged as an activity that could fit into routine reviews in social care or primary care. This use was felt by some to constitute an intervention, as it would shift the focus of care by virtue of asking holistic questions about LTCs. Interviewees suggested that a PROM would help to capture the patient's perspective quickly and effectively.

#### A means of involving patients in their own care

The use of a PROM to enable the active involvement of patients in their own care was strongly supported by the majority of participants, but some participants were uncertain around how best to achieve this type of involvement.

It was felt that a PROM could facilitate patient empowerment, as it would encourage patients to reflect on their progress and receive information back on how they were doing in comparison to others, and with their own previous scores. Feeding information into services about their outcomes could also enable patients to shape and affect service provision. Similar to the second sub-theme above, it was argued that this use could be linked to a regular event, such as a routine review, but there was also a contingent arguing that patients might want the flexibility to use an LTC PROM at any desired time.

#### Capturing the outcomes of interventions

A significant number of participants (n=14) contended that PROM use should be linked to specific services or interventions for patients, so that before–after PROM scores could be captured. This differed from other proposed uses by positioning the PROM as a measurement tool rather than a form of intervention in and of itself.

While some suggested that an LTC PROM could be employed to capture the outcomes of any identifiable intervention or treatment change, most participants who suggested this use specifically recommended linking the PROM to care planning. As care planning explicitly aims to involve patients in their care decisions, a PROM was argued to be a useful complementary tool for enabling involvement (by asking patients to reflect on their conditions), and for tracking the success of care planning (by recording progress in terms of outcomes).

#### Using a PROM for multiple purposes

Typically, front-line clinicians and those with a commissioning and/or policy role both wanted a PROM to work on the individual and aggregate level, but front-line clinicians most strongly advocated individual use. Only one regulator (P14) and one public health commissioner (P18) solely advocated PROM use at the aggregate level.

The ideal promoted by most participants was that the PROM should be feasible and useful at the individual level, first and foremost, with any aggregate use being an additional benefit. Focusing on the individual level meant that stakeholders prioritised the usability of the PROM for individuals engaged in managing care (patients and practitioners) over its broader applicability. Participants indicated that they would prefer a PROM if it could both inform individual care and help improve services in general.

### Theme 2: concerns around PROM use and implementation

Despite strong support for the notion of an LTC PROM, there were a number of concerns about its feasibility and practical application. The three main concerns are outlined below (see also table 3). These concerns revolved around how to ensure that meaningful PROM data could be collected and shared efficiently (PROM implementation: engaging patients and practitioners and PROM implementation: divisions across services), and how to ensure that PROM data are used in intended and appropriate ways (PROM use: interpretability and usability of PROM data). These concerns reflected general agreement that an LTC PROM would be valuable if implemented and interpreted carefully.

**Table 3:** Theme 2—concerns around PROM use and implementation

Theme	Subthemes	Examples
Concerns around PROM use and implementation	PROM implementation: Engaging patients and practitioners	If we're talking about clinicians (…) [they] need to feel ownership of the measures they use—they need to feel that, you know, I'm using this because I feel it's the right thing to do; I'm convinced by its validity and I think it works with my patients (P 20, Voluntary Organisation)
		Part of the buy-in is to get the patients to take control of it and feel like it's useful to them first (…) if it's seen as some kind of measure of them at a point in time, they might think it was, you know, it could be used against them or it might be used to justify doing or not doing something that they want to do (P29, GP)
		There's no good me handing a questionnaire to a patient in a meeting asking them to rate the quality of the service I'm now giving them (…) and asking them to hand it back to me, that's not going to work (…) If it's something about am I [the patient] actually achieving some of my goals and you've got a properly collaborative relationship with them, that shouldn't be a problem (P30, Healthcare Provider)
	PROM implementation: Divisions across services	We happen to historically have built a wall around something we call health (…) And we've built a wall around something that we call social care (…) [but] if we're being person centred, we want to understand all of those domains around [people with LTCs] and to think about how that…how support to that individual can be provided and that will then involve relationships between things—services—which we have compartmentalised (P8, Voluntary Organisation)
		What would be really nice would be if barriers between the different organisations that look after people with long-term conditions were easier to overcome (P11, GP)
		The issues that we've encountered with social care and health mixing is boundaries really (P29, GP)
	PROM use: Interpretability and usability of PROM data	There needs to be a set of principles, there needs to be an agreement, there needs to be some sort of broader oversight around all of that [interpretation] because lots of different parts of the system will want to use the data (…) I think there needs to be an agreement about how we manage the analysis and the interpretation (P14, Regulator)
		Interpretation of any data has to sit within a wider understanding of what's going on because reported measures in any way can be misinterpreted (P25, Social Care)
		A measure may be designed for a purpose but if the beliefs and behaviours of the people in the system are driven by a different purpose (…) then that measure will be captured and re-interpreted into that purpose (P9, GP and Voluntary Organisation)
		Unless people understand the context of the data (…) you can make sweeping assumptions about the data (P30, Healthcare Provider)
		You have to be really sure that it's doing the job you want to do and not just becoming a reporting measure within the, you know, for instance within the commissioning system (Participant 20, Voluntary Organisation)

GP, general practitioner; LTC PROM, long-term conditions patient-reported outcome measures; NHS, National Health Service.

#### PROM implementation: engaging patients and practitioners

Participants stressed that patient and practitioner both needed to perceive the value of a PROM and feel some ownership over the process of using PROM data. It was felt that an LTC PROM would need to be implemented in the context of a collaborative, person-centred relationship between patient and practitioner, in order to produce useful data for patients, practitioners and services.

Several interviewees (n=12) pointed to cultural barriers in current practices that did not necessarily support working with patients in a person-centred way, for instance, a focus on biomedical indicators in primary care reviews, or a need to financially ration services. Another potential barrier was pre-existing expectations within the patient–practitioner relationship. Interviewees felt that patients might feel uncomfortable criticising services directly, or be concerned about how their answers would influence future access to services. Similarly, concerns were raised that practitioners would find it difficult to modify their practice to treat patients as equal partners in decision-making. However, it was believed that an LTC PROM as a tool could help to promote a more person-centred mode of practice, by bringing in an explicit focus on measuring what matters to the patient and facilitating joint decision-making based on PROM data.

Another challenge participants perceived was balancing the priorities and needs of the different stakeholders when implementing a PROM in practice. They argued that care needed to be more person-centred, and hoped that a PROM would help, but worried about how to resolve differences between what patients prioritised and what practitioners prioritised.

#### PROM implementation: divisions across services

In line with current policy intentions, participants were keen on the idea of a measure that might aid greater integration across all health and social care services. However, they foresaw difficulties related to data sharing across services, where traditional division of responsibilities could create barriers. It was felt that practitioners could be reluctant to work outside the boundaries of their current role, due to pressure around achieving service-specific targets. Interviewees felt that services needed to be shifted towards more integrated modes of working, and that a shared outcome measure could form part of this shift, but that without systemic support, integrated working would struggle.

#### PROM use: interpretability and usability of PROM data

Related to the issue of engagement, participants talked about the need for a clear set of principles or standards for analysing, interpreting and using PROM data. Interviewees were concerned that PROM data would not influence practice or commissioning decisions if the means to interpret the data were not transparent and agreed on by stakeholders.

Participants also worried about PROM data being misunderstood, over-interpreted or used inappropriately. They argued that PROM data should be contextualised and carefully interpreted in light of the purpose for collecting the data and the circumstances of collection.

Interviewees made two suggestions on how to ensure the interpretability and usability of PROM data. The first suggestion was to link the PROM to measuring outcomes for a specific intervention; the second, to focus on ensuring usability at the clinical level for the patient and practitioner.

### Theme 3: content of an LTC PROM

Participants discussed a number of potential domains or items that should be included if a new PROM were to be developed. Key topic areas endorsed by participants are outlined in tables 4 and 5 and are described below.

**Table 4:** Theme 3—content of an LTC PROM

Theme	Subthemes	Examples
Content of an LTC PROM	*Endorsement of Domains*	*See* table 5 *for detailed account*
	Shared outcomes across LTCs	You have to look at people holistically and think about what's important to them about, you know, their activities, the daily living and how well or not they're able to perform those to whatever degree is acceptable to them (P2, NHS Commissioning)
		We think that actually most people with long-term conditions have eighty percent of their support needs as being general, not condition specific (P7, NHS Policy and Commissioning)
	The place of process/experience domains	So what's your outcome for me would mean, are you getting the right treatment for whatever it is, or the right services for whatever it is; is it meeting the outcomes that you want as a person; are you having a positive experience of all of that and does it feel safe (P7, NHS Policy and Commissioning)
		[A PROM would] be easier to normalise if it's combined with some experience measures at the same time (P19, Healthcare Provider)
		I would fairly argue that experience is an outcome (…) and also patient experience is linked to other outcomes (…) So I would say they are all part of one sort of view on what good quality looks like. So I wouldn't want to separate experience out of outcomes (…) I wouldn't want it to be seen as a less important part of how you measure a good outcome (Participant 28, Voluntary Organisation)
	Importance of stakeholder involvement in PROM design	I would hope that any PROM development is done, you know, including clinicians, but also including the patients who are expert in their own ways about what their symptoms are and how they can be managed most effectively (P2, NHS Policy and Commissioning)
		[We need] more direct involvement of people in [PROM] development (…) once it actually goes out into the real world we have to have complete confidence that it's relevant to the people and it reflects what they think (P16, Voluntary Organisation)
		If you're going to design something you need to talk to the people who would be affected and really get their views on it (P21, Social Care)

GP, general practitioner; LTC PROM, long-term conditions patient-reported outcome measures; NHS, National Health Service.

**Table 5:** Endorsement of domains for a PROM for LTCs

Domains	Endorsement	Quotes
Empowerment	23 interviews	In terms of getting patients to participate in their care and to understand what matters to them, then we need to be measuring that because if we're not we're not going to change the way we do things (*Participant 3, NHS Policy and Commissioning*)
		If it's a question about how in control the patient feels then that's great. If it's a question that says something like, 'Do you feel you're able to self-manage? ’ I'm not sure how well I would be able to answer that as a patient (*Participant 1, GP*)
		When you listen to people you know they talk about being in control, wanting to have the information to be in control of their life (…) that's very much related to health (*Participant 6, GP and CCG*)
		We need to build a more nuanced framework that takes into account the personal goals and the empowerment of the individual as well (*Participant 12, NHS Policy and Commissioning*)
		I'm thinking there should be some consistent ones [items] (…) because they
		are about resilience, ability to cope, self-care, confidence, you know, regardless of what you've got (*Participant 25, Social Care Commissioner*)
Quality of life or impact of illness and/or treatment on life	17 interviews	For long-term conditions measuring around, or focusing around sustainability of where they are and, I guess a bit around their quality of life and experience as well as actual clinical outcomes would be the thing to do (*Participant 19, Healthcare Provider*)
		I think we need to understand what the impact is of quality of life (…) through engagement with the system (*Participant 14, Regulator*)
		I think you want to know how the condition affects their daily life, that's a pretty obvious one (…) I think to what extent it affects their daily life and how important it is to them (*Participant 11, GP*)
Patient-specific or personalised goals	14 interviews	It's the outcomes that are important to me [the patient] *(Participant 20, Voluntary Organisation)*
		I think there needs to be something in there around…you know what is the outcome…am I getting outcomes in terms of my, you know, my goals. Am I getting outcomes in terms of how I want my care to be done? (*Participant 3, NHS Policy and Commissioning*)
		It's how you get beyond those biomedical outcomes to decide what is the main thing that matters to the patient really (*Participant 24, Public Health*)
		As long as it was in a framework, people would get to personalise within a framework (…) I'd pick the ones [outcomes] that are most relevant to them (*Participant 25, Social Care*)
Functioning (including social, physical and psychological)	14 interviews	A focus on function and functioning is much more important, and actually maybe that helps more [with] multimorbidities (*Participant 1, GP*)
		You could think about it from a sort of motor sensory affective and functional domains [perspective] (…) and then subdivide them potentially. I suppose it could be…I think certainly having an affective domain would be useful and having a functional one would be useful and I think that whether you drilled down to very specific things…I don't know I guess it would depend on the condition (*Participant 29, GP*)
		I guess if you're trying to do generic long-term conditions, I'm kind of interested in
		well-being, functional status and probably pain (*Participant 4, GP and CCG*)
		Social isolation is one [outcome of interest] for us, as is how mobile people are, so [is] how self-sufficient they are (*Participant 26, Social Care Commissioner*)
Social participation	13 interviews	I welcome something about social participation, that's really important (*Participant 6, GP and CCG*)
		Many of the people I work with in mental health, what they want to focus on is having a roof over their head, having some money coming in and having some friends (…) we need to see what we're doing around that and that quality of life and that…all the stuff around social inclusion (…) and are we meeting what the patient wants (*Participant 30, Psychiatrist*)
Psychological well-being	11 interviews	[Currently] a lot of things that we capture tend to be just focusing on the physical health, and as a matter of routine what we want to try and change is that actually people's mental well-being is considered in terms of some of the core questions asked (*Participant 7, NHS Policy and Commissioning*)
		Mental health well-being is something that could be common across them all [long-term conditions] (*Participant 23, Nurse Practitioner*)
Symptoms or clinical outcomes	7 interviews	I think pain is a key issue and that, you know, the management of pain (*Participant 20, Voluntary Organisation*)
		For long-term conditions measuring around, or focusing around sustainability of where they are and, I guess a bit around their quality of life and experience as well as actual clinical outcomes would be the thing to do (*Participant 19, Healthcare Provider*)
Access to services (includes access to information)	5 interviews	I mean it's not really an outcome measure but in terms of people accessing services I thought that one thing that could be common across all [conditions] is any frustrations that people might feel, which then in turn affects their self-esteem or their self-empowerment, (…) if they can't get the service or the medication they need (*Participant 23, Nurse Practitioner*)
Joined up nature of services	5 interviews	The patient reported outcome is that their care feels joined up (…) but you'd have to word it differently to make it a PROM rather than a PREM [Patient-Reported Experience Measure] (*Participant 10, Consultant*)
Impact on carers	4 interviews	Part of the one lens for a PROM is how well does my care support…how well are my carers supported with me in getting my best possible outcome (*Participant 12, NHS Policy and Commissioning*)

GP, general practitioner; LTC PROM, long-term conditions patient-reported outcome measures; NHS, National Health Service.

#### Shared outcomes across LTCs

Participants suggested a PROM for LTCs that would focus on traditional health-related outcomes, such as quality of life, mental well-being and physical functioning, with the inclusion of less traditional outcomes such as empowerment and social participation.

The majority endorsed domains that capture the role of the patient in managing their own LTC(s) and the role of services in enabling or supporting self-management. This was variously described as ‘empowerment’, ‘activation’, ‘supported self-management’, ‘self-control’, ‘self-reliance’, ‘knowledge, skills and confidence to manage’, ‘being a partner in their care’, ‘feeling informed and in control’ and ‘control over daily life’ (see table 5 for examples). Across health and social care settings, interviewees stressed the importance of supporting people to develop their knowledge, ability and confidence in managing their own health.

Most participants felt that similarities in the management goals for different LTCs outweighed the differences. Similarly, interviewees tended to agree that a shared measure across health and social care services was viable, if the content was generated in collaboration with patients and practitioners.

#### The place of process/experience domains

Participants were divided over the extent to which people's experience of using services should be included in an LTC PROM. Some saw experience of services as another form of outcome; others saw it as an important part of the process that could influence outcomes, but not as an outcome in its own right. Some participants felt that incorporating experience-related items could lead to the PROM being used to meet performance targets.

What most people agreed on was that there were important experience domains (such as access to services, information and care coordination) that need to be measured in some way as part of improving commissioning and provision of care. Some participants argued that combining experience and outcome measurement would aid integration into practice and ensure a measure was more meaningful to patients.

In general, it was felt that experience-related domains could be part of an LTC PROM so long as they mattered to patients, and the context of data collection was taken into consideration when designing and implementing the PROM.

#### Importance of stakeholder involvement in PROM design

While participants endorsed a number of topic areas as relevant for LTCs, they unanimously agreed that patients and practitioners needed to be involved in determining which domains should be included. Participants also agreed that patients and practitioners should both be involved in decision-making around how to implement and use an LTC PROM.

Interviewees were clear that an LTC PROM should be designed in collaboration with its end-users (especially people with LTCs and front-line practitioners), to ensure that it captures the domains they value, and that it is feasible and acceptable for use.

## Discussion

This study incorporates perspectives on the value of an LTC PROM from health, social care and voluntary organisations and managerial, policy and front-line levels. We found broad support for the idea of a PROM that could be used by people with a wide range of LTCs in various settings. Stakeholders particularly identified three main uses for an LTC PROM: to inform individual care; to encourage patient involvement in their own care; and to monitor and evaluate services based on shared outcomes. While the focus of this study was on the health and social care system in England, the issues discussed are likely to apply to other contexts and countries facing the challenge of improving LTC care.^7–9^

Use at the individual level was prioritised over use to inform population-level service monitoring. Most PROMs have been designed to work at an aggregate rather than individual level, and can be of limited value for individual level use.^44^ Developing and validating an instrument for use at the individual level will be challenging, as the measure will need to be more precise to capture meaningful change at an individual level.^45^ It is worth noting that while application at the individual level was preferred, most interviewees still desired a PROM that would enable comparisons across groups, rather than a completely personalised measure.^46^

Stakeholders endorsed a broad range of traditional as well as non-traditional domains, such as functioning, quality of life, empowerment, social participation, the experience of services and feeling supported by services. These domains reflected a shared understanding of the direction in which LTC care needs to move, that is, towards person-centred care, a holistic and integrated approach to patient needs and greater patient involvement in care decisions and support for self-management. The unanimous insistence that patients and front-line practitioners be involved in designing the content of an LTC PROM also reflected the emphasis on greater patient involvement in LTC care. The suggestion to include particular experience domains was of note; experiences and outcomes are typically measured separately, or are measured by adding an existing PROM into an experience survey.^39^ It would clearly be more efficient for services if all relevant outcomes and experience data could be gathered at the same time, as experience data could inform service quality while outcome data help establish effectiveness.^47^ However, outcomes and experience of services have different implications for when, where and how to collect data, that need to be further explored.

Interviewees highlighted concerns and challenges that reiterate findings in previous studies with primary and secondary care clinicians.^48–50^ Concerns revolved around the feasibility of implementing a PROM in routine care, particularly in relation to administration, interpretation and application. It was acknowledged that a PROM could not only measure and record outcomes but also be used as a mechanism to change the focus, content and process of LTC care.

Implementing a PROM in any setting is not simple; it could be more accurately described as a complex intervention.^51^ Attempts to implement PROMs in clinical practice have achieved mixed results, with factors at all stages impacting on success.^14^ ^16^ ^25^ ^26^ ^49^ ^52^ ^53^ Collecting local or national PROM data is feasible; achieving changes based on these data has been less successful.^19^ ^21^ Two interlinked issues need to be addressed in order to achieve successful implementation and use of an LTC PROM.

First, given the scope of topics suggested for an LTC PROM, further research will be required to design a measure that can succinctly capture key shared outcomes across LTCs. Interviewees stressed the importance of determining PROM content with PROM end-users—this would require engaging patients using health and/or social care, identifying and engaging specific services in which the PROM would be used and engaging practitioners who may use PROM data. Establishing PROM content and purpose/s for specific contexts would best work in tandem, and would inform practical and methodological decisions around modes of data collection, analysis and feedback.^54^ One potential purpose identified in the study was to develop a PROM as a mechanism for engaging patients in self-monitoring outcomes as identified through care planning.^55^ The feasibility of engaging patients in using PROMs to guide their own care will need investigation; though patients tend to be positive about PROMs, few studies have explored actively involving patients in interpreting and using PROM data.^13^ One way to proceed is to adopt a user-focused approach to design: establishing and refining the content of a draft measure in consultation with patients and practitioners, making decisions as to what to include or exclude based on feasibility and the end-users’ priorities for use, and finalising the measure's content and format following pilot testing in specific contexts. This process may lead to some uses being prioritised over others.

Second, organisational and cultural barriers will be significant challenges. In order to implement an LTC PROM, organisational support for the process of collecting, analysing and using these data needs to be established.^56^ PROM programmes such as WestChronic in Denmark have achieved success due to the principle behind PROMs being supported at an organisational level, demonstrated through integration of PROMs into the clinical infrastructure. Hjollund *et al*^22^ argue that this is more likely to be achieved if data can be used at both the individual and organisational level. In addition, a significant factor in integrating PROM data into practice is the cultural value attached to the patient-centred approach.^22^ ^56^ A combination of education, engagement and mutual negotiation with practitioners, alongside organisational support, is likely to be needed in order to demonstrate the value of the patient-centred approach, and the value of PROMs as a patient-centred tool that can complement existing practice.^57^

This suggests an approach that incorporates a rigorous development of content involving all the potential end-users of the PROM, including patients,^58^ with an equally rigorous understanding and evaluation of the contexts for use,^59^ and the potential mechanisms for change.^12^ ^13^ This is likely to require an iterative process of development and theory-driven implementation, working closely with front-line practitioners and patients, and using mixed methods research to evaluate the context, process and outcomes of PROM use.^12^ ^15^ ^60–63^ Clearly, an essential first step would be to define meaningful outcomes for LTCs with patients, and establish ways in which they would value using and sharing this information.

### Strengths and Limitations

It is a strength of the study that it incorporated a broad range of perspectives across health and social care, but it should be acknowledged that some subgroups were under-represented (such as practice nurses or front-line social workers). In addition, as some participants entered the study via snowball sampling technique, this may have led to a bias towards people who share similar ideas taking part. Practitioners who took part had some level of interest in PROMs and LTCs, and so may not be typical of all front-line practitioners. Patients’ views were not captured in this study, but will be the focus of the next phase of research.

## Conclusion

Stakeholders from a range of backgrounds in health, social care and voluntary organisations were supportive of a PROM that would work across LTCs, and valued the idea of a PROM that could be used to improve care at the individual clinical level. Stakeholders endorsed an LTC PROM that captured traditional as well as non-traditional domains, such as functioning, quality of life, empowerment and social participation and recommended that patients be involved in its design. In order to achieve the goals outlined by stakeholders, designing and implementing an LTC PROM will require engaging the potential end-users of the PROM and the organisations within which the PROM will be used.
